# Differentiated Responses of Apple Tree Floral Phenology to Global Warming in Contrasting Climatic Regions

**DOI:** 10.3389/fpls.2015.01054

**Published:** 2015-12-15

**Authors:** Jean-Michel Legave, Yann Guédon, Gustavo Malagi, Adnane El Yaacoubi, Marc Bonhomme

**Affiliations:** ^1^INRA, Unité Mixte de Recherche 1334 Amélioration Génétique et Adaptation des Plantes Méditerranéennes et TropicalesMontpellier, France; ^2^CIRAD, Unité Mixte de Recherche 1334 et Inria, Virtual PlantsMontpellier, France; ^3^Faculdade de Agronomia, Universidade Federal de PelotasPelotas, Brazil; ^4^Faculté des Sciences, Université Moulay IsmailMeknès, Morocco; ^5^Unité Mixte de Recherche 547, INRA et Université Blaise Pascal, PIAFClermont-Ferrand, France

**Keywords:** fruit tree, flowering, chill period, heat period, warming vulnerability, multiple change-point models

## Abstract

The responses of flowering phenology to temperature increases in temperate fruit trees have rarely been investigated in contrasting climatic regions. This is an appropriate framework for highlighting varying responses to diverse warming contexts, which would potentially combine chill accumulation (CA) declines and heat accumulation (HA) increases. To examine this issue, a data set was constituted in apple tree from flowering dates collected for two phenological stages of three cultivars in seven climate-contrasting temperate regions of Western Europe and in three mild regions, one in Northern Morocco and two in Southern Brazil. Multiple change-point models were applied to flowering date series, as well as to corresponding series of mean temperature during two successive periods, respectively determining for the fulfillment of chill and heat requirements. A new overview in space and time of flowering date changes was provided in apple tree highlighting not only flowering date advances as in previous studies but also stationary flowering date series. At global scale, differentiated flowering time patterns result from varying interactions between contrasting thermal determinisms of flowering dates and contrasting warming contexts. This may explain flowering date advances in most of European regions and in Morocco vs. stationary flowering date series in the Brazilian regions. A notable exception in Europe was found in the French Mediterranean region where the flowering date series was stationary. While the flowering duration series were stationary whatever the region, the flowering durations were far longer in mild regions compared to temperate regions. Our findings suggest a new warming vulnerability in temperate Mediterranean regions, which could shift toward responding more to chill decline and consequently experience late and extended flowering under future warming scenarios.

## Introduction

Phenological events are highly responsive to temperature (Menzel and Fabian, 1999) and the abundance of information on plant phenology outlined substantial responses to global warming (Rutishauser et al., 2009). Most studies focused on bud phenology in natural vegetation and exhibited flowering advances as a main warming response (Abu-asab et al., 2001). Concerning fruit trees, flowering advances were highlighted in the European warming context for apple, pear and cherry trees (Chmielewski et al., 2004; Guédon and Legave, 2008; Eccel et al., 2009), hazelnut tree (Črepinšek et al., 2012), and olive tree (Garcia-Mozo et al., 2009). This was also observed in the Northeastern American context for apple tree (Wolfe et al., 2005) and in various parts of Asia for cherry tree (Miller-Rushing et al., 2007), apple tree (Fujisawa and Kobayashi, 2010), chestnut tree (Guo et al., 2013), and citrus species (Fitchett et al., 2014). Similar flowering advances have been scarcely reported in the Southern Hemisphere for apple and pear trees (Grab and Craparo, 2011).

Moreover, several studies dealing with warming responses in various perennial plants grown in temperate conditions found stationary or delayed bud phenology, in spite of temperature increases (Doi and Katano, 2008; Gordo and Sanz, 2009; Schwartz and Hanes, 2010; Yu et al., 2010). In fact, the timing of flowering is controlled by multiple and complex determinisms related to temperature at different periods of the year (Cook et al., 2012; Guo et al., 2013). Most temperate trees, including fruit species, are dormant in autumn and winter. Since the work of Lang et al. (1987), it has been widely accepted that among the different phases of bud dormancy, endodormancy corresponds to the growth suspension of the meristematic activity. The dormant buds require exposure to chill temperatures in order to overcome the endodormancy phase, followed by exposure to heat temperatures to resume growth during an ecodormancy phase and to initiate flowering in spring (Campoy et al., 2011). One likely warming impact during endodormancy is a delay in the fulfillment of chill requirements and consequently a delay in the time at which perennial plants become receptive to heat temperatures (Yu et al., 2010; Luedeling et al., 2013). This may explain unexpected phenological changes like those observed in walnut trees grown in California for which the vegetative buds (high chill requirements) shifted to late leaf-out since 1994 (Pope et al., 2013). Inversely, the flowering advances, that have dominated climate-warming responses thus far, were explained by increasing temperatures during ecodormancy leading to a more rapid fulfillment of heat requirements, as shown for apple trees in Europe (Legave et al., 2013) and in Japan (Fujisawa and Kobayashi, 2010). A comprehensive assessment of divergent responses to warming in temperate perennial plants must thus include the potential impacts on the fulfillment of both chill and heat requirements (Schwartz and Hanes, 2010). The sequential chill-growth model was therefore commonly used for analyzing flowering times in temperate fruit trees (Eccel et al., 2009; Darbyshire et al., 2014). When the fulfillment of chill requirements is inadequate, as is currently the case in mild climates, a typical symptom is the extended duration of the flowering phase (Atkinson et al., 2013). However, less attention has been paid to change in flowering duration in response to climate warming (Miller-Rushing et al., 2007; Legave et al., 2013). Moreover, in the case of fruit trees, nearly all the studies have reported warming responses in only one location or a few locations submitted to similar climatic contexts (Chmielewski et al., 2004; Fujisawa and Kobayashi, 2010; Grab and Craparo, 2011; Črepinšek et al., 2012), whereas it has been demonstrated that a given species can have contrasting responses in different locations (Primack et al., 2009). As an illustration, a large spatially-distributed lilac data set in North America demonstrated that the floral phenology has progressively changed from advances in flowering in northern regions to delays in flowering in southern regions (Zhang et al., 2007). In fact, there is evidence that more field studies are needed to determine the extent to which phenological shifts are occurring on large geographical scales (Primack et al., 2009).

Another key question is the use of appropriate statistical methods for analyzing flowering date and temperature series. The statistical analysis of such series is not standardized and various methods were used including linear regression (Fujisawa and Kobayashi, 2010; Grab and Craparo, 2011), multiple change-point models (Guédon and Legave, 2008) and segmented regression models (Pope et al., 2013). Compared to our previous study (Guédon and Legave, 2008), we extended in this study the statistical modeling framework in order to test not only piecewise constant models but also piecewise linear models that include simple linear regression models when no change point can be detected. We were thus able in this way to identify both abrupt changes and linear trends in phenological series.

Our objectives here were (i) to propose a statistical modeling framework for analyzing flowering date and temperature series with minimum a priori assumptions (ii) to identify on this basis differentiated flowering changes on a large geographical scale in apple tree and (iii) to understand how changing temperature conditions can lead to differentiated flowering changes. These complementary objectives included changes both of the flowering time and the flowering duration. Apple tree offers a relevant study plant because of its worldwide cultivation and relatively high chill requirements (Hauagge and Cummins, 1991a; Ghariani and Stebbins, 1994) which can result in divergent responses to change in temperature conditions (Schwartz and Hanes, 2010).

## Materials and methods

### Flowering and temperature data

#### Collection of flowering date series

**Figure 1:**
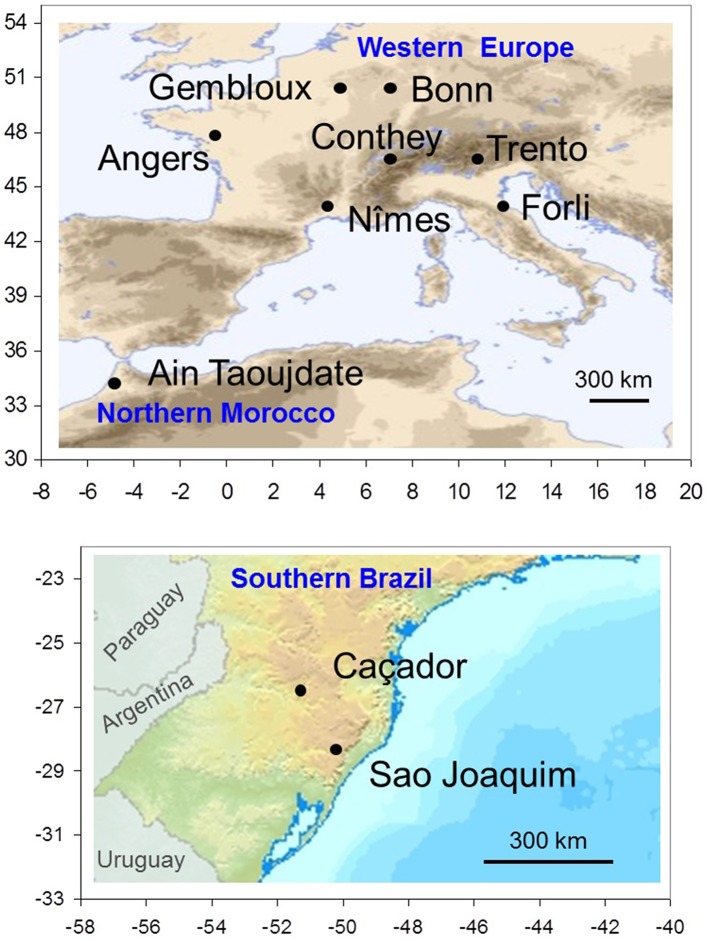
**Spatial distribution of locations where flowering and temperature data were collected: eight locations in the Northern Hemisphere (upper map) and two locations in the Southern Hemisphere (lower map)**. The ranges of longitude and latitude are reported in abscissa and ordinate.

A collaborative international network on apple tree phenology has been established between research institutes in six countries. We selected 10 locations, seven in Western Europe, one in Northern Morocco and two in Southern Brazil (Table 1; Figure 1). The eight locations in the Northern Hemisphere are located across a large latitudinal range (from 34 to 50°N) with a corresponding large range of climatic conditions during the dormancy and flowering phases, from a cold continental climate in Europe (Bonn, Gembloux, Conthey, Trento) to a mild climate in Northern Morocco (Ain Taoujdate). This includes European locations with intermediate climates such as oceanic (Angers) and Mediterranean (Forli, Nîmes). While situated at high elevation to favor apple cropping, the two Brazilian locations in the Southern Hemisphere are clearly characterized by mild climates during the dormancy and flowering phases (mean temperature up to 11°C).

**Table 1:** **Description of the collected data**.

**World region *Location***	**Latitude/Longitude**	**Elevation (m.a.s.l.)**	**Climatic conditions *Climatic influence***	**Temperature data period**	**Phenological data**			**Collaborative institute**
					**Period**	**Cultivar**	**Stage (BBCH)**	
Western Europe			Temperate					
*Bonn, Germany*	50.62/6.98	160	*Continental*	1959–2013	1958–2013	Golden D.	61,65	INRES
*Gembloux, Belgium*	50.57/4.68	138	*Continental*	1964–2013	1984–2013	Golden D.	61	CRA-W
*Angers, France*	47.47/-0.63	38	*Oceanic*	1963–2013	1963–2013	Golden D.	61	INRA France
*Conthey, Switzerland*	46.22/7.30	504	*Continental*	1970–2013	1970–2013	Golden D.	65	Agroscope
					1975–2013	Gala	65	
*Trento, Italy*	46.07/11.12	419	*Continental*	1983–2013	1983–2013	Golden D.	61,65	CRA-FRF
*Forli, Italy*	44.22/12.03	34	*Mediterranean*	1970–2013	1970–2013	Golden D.	61,65	CRA-FRF
*Nîmes, France*	43.73/4.50	52	*Mediterranean*	1966–2013	1974–2013	Golden D.	61,65	Ctifl
					1979–2013	Gala	61,65	
					1980–2013	Fuji	61,65	
Northern Africa			Mild					
*Ain Taoujdate, Morocco*	33.93/−5.22	499	*Mediterranean*	1973–2013	1984–2013	Golden D.	61,65	INRA Morocco
*Southern Brazil*			*Mild*					
*Caçador, Santa Catarina*	−26.78/−51.02	960	*Subtropical*	1961–2013	1984–2013	Golden D.	61,65	EPAGRI
					1982–2013	Gala	61,65	
					1982–2013	Fuji	61,65	
Sao Joaquim, Santa Catarina	−28.29/−49.93	1353	*Subtropical*	1955–2013	1972–2013	Golden D.	61,65	EPAGRI
					1972–2013	Gala	61,65	
					1976–2003	Fuji	61,65	

Within this extensive geographical area, flowering dates were recorded for the beginning of the bloom phase (~10% of flowers open) and the full bloom (~50% of flowers open, first petals may have fallen). These dates correspond to stages 61 and 65 of the international BBCH code, respectively. Experienced observers recorded them using similar observation procedures on adult trees grown in long-term orchards. At each location, the flowering dates were assessed at least twice weekly on several trees of a given cultivar. In the mild conditions of Morocco and Brazil where flowering duration is extended (see Results) and flowering intensity is frequently weak due to floral abortions (Oukabli et al., 2003; Petri and Leite, 2004), the observers were trained to collect accurate data for comparison with those collected in temperate conditions. New trees were observed periodically at all locations as trees aged, whereas new observers were trained by the preceding ones.

To compare long-term flowering series between different locations, we chose cultivars grown worldwide. We therefore collected numerous data for Golden Delicious for which records were available at all 10 locations. In addition, records for Gala and Fuji were collected since these cultivars were frequently grown in Southern Brazil, but also in Europe. These three cultivars were characterized by nearly the same high chill requirements (Hauagge and Cummins, 1991a) and concomitant flowering times both in Southern Brazil and Europe (cross pollination in orchard). The collection of different varietal series in a given location was thus considered as a way to repeat the statistical analysis to reveal a strong phenological change in the location, and not as a way to study the genotype × location interactions. Our data set consists of 30 flowering date series including series for the stages 61 and 65 (16 of them corresponded to the temperate conditions and 14 to the mild conditions). Each series was defined by a location, a cultivar and a flowering stage, including a total of 1121 measurements. Most series were complete aside from some missing data (not interpolated) in some series. The longest series contains 56 years in Bonn (Golden Delicious, stages 61 and 65) and the shortest contains 25 years in Caçador (Golden Delicious, stages 61 and 65; Table 1). The consistency of collected data was assessed by the fact that the flowering dates were consistently related to the geographical characteristics (latitude, elevation) and temperature conditions of the locations. Moreover, the flowering duration between the dates of stages 61 and 65 was assessed at all locations where the two dates were recorded. This included 13 series of flowering durations (six for the temperate conditions and seven for the mild conditions) ranging from 56 years in Bonn (Golden Delicious) to 25 years in Caçador (Golden Delicious).

#### Collection of temperature series

For characterizing the relationships between flowering and temperatures, we analyzed series of mean temperatures during two successive periods respectively determining for the fulfillment of chill and heat requirements. Annual chill accumulation (CA) period and subsequent heat accumulation (HA) period have thus been defined. Based on previous results concerning the bud dormancy dynamics (Malagi et al., 2015) and the relationships between flowering and temperatures (Legave et al., 2013; El Yaacoubi et al., 2014) in apple tree, the CA period ranged from October to January for the European and Moroccan locations (Northern Hemisphere) and from April to July for the Brazilian locations (Southern Hemisphere). The HA period ranged from February to April for the European locations and from August to October for the Brazilian locations. We chose a shorter HA period for the Moroccan location (March to mid-April), because previous works using Partial Least Squares regression clearly suggested this period as a major period of heat requirement fulfillment in Morocco (El Yaacoubi et al., 2014).

The mean temperature series were constituted from the average minimum and maximum daily temperatures collected from weather stations located near the orchards where the flowering dates were recorded (no more than 10 km). The daily temperatures were provided for each location and checked by the corresponding research institute. The French partner performed a complementary global check for this study. The few missing data were estimated by linear interpolation. All the series started before the end of the 1980s, the instant at which marked increases of temperature have been frequently recorded at the world scale, particularly in Europe (Jones and Moberg, 2003). When temperature series longer than the corresponding flowering date series were available, we collected the longest possible temperature series; this was the case for Gembloux, Nîmes, Ain Taoujdate, Caçador, and Sao Joaquim (Table 1).

### Statistical modeling

#### Definition of piecewise constant and piecewise linear models

Multiple change-point models were used to delimit segments within a flowering date or temperature series of length *T*, for which the data characteristics were homogeneous within each segment while markedly differing from one segment to another. We made the assumption of homoscedastic Gaussian multiple change-point models, either piecewise constant or piecewise linear models. In the first case, the slope is assumed to be zero and the only within-segment parameter is the intercept (which is also the segment mean in this case) whereas in the second case, the within-segment parameters are the intercept and the slope. In both cases, the variance is assumed to be common to the segments. This homoscedasticity assumption is justified by the data characteristics but also by the fact that the series were rather short (between 25 and 56 years). The two associated models are denoted by *M*_constant_ (for piecewise constant) and *M*_linear_ (for piecewise linear). Piecewise linear models are somewhat related to the segmented regression models used by Pope et al. (2013). Segmented regression or broken-line models are regression models where the regression function is piecewise linear, i.e., made of straight lines connected at change points (Muggeo, 2003). The regression function is thus continuous, but first derivatives are discontinuous. In our case, the regression function is not constrained to be continuous.

For the *M*_constant_ model, we suppose that some *J* − 1 instants τ_1_ < ⋯ < τ_*J* − 1_ (with the convention τ_0_ = 0 and τ_*J*_ = *T*) exist such that the mean is constant between two successive change points and the variance is assumed to be constant,

(1) $$\text{if }\tau_{j}\leq t<\tau_{j\;+\;1},\;\{\begin{array}{c} E(X_{t})=\alpha_{j},\\ \text{Var}(X_{t})=\sigma^{2}. \end{array}$$

These two families of models enable to test and combine two assumptions: change point of sufficient amplitude separating two phases and linear trend (within phase or for the whole series in the case of no change point).

We adopted a retrospective or off-line inference approach whose objective was to infer the number of segments *J*, the instants of the *J* − 1 change points τ_1_, …, τ_*J* − 1_, the *J* within-segment intercepts α_*j*_, the global variance σ^2^ and the *J* within-segment slopes β_*j*_ (for *M*_linear_ model). For the selection of the number of segments *J*, we used the modified Bayesian information criterion (mBIC) proposed by Zhang and Siegmund (2007) and specifically dedicated to Gaussian homoscedastic multiple change-point models. The principle of this kind of penalized likelihood criterion consists in making a trade-off between an adequate fitting of the model to the data and a reasonable number of parameters to be estimated. Jeffreys' rules of thumb (Kass and Raftery, 1995) suggest that a difference of mBIC of at least 2 log(100) = 9.2 is needed to deem the model with the higher mBIC substantially better. For the optimal segmentation of the series into *J* segments, we applied the dynamic programming algorithm proposed by Auger and Lawrence (1989). This optimal segmentation defines the optimal change points and relies on the estimation of within-segment and global variance parameters; see details on these statistical methods for multiple change-point models in Supplementary Material, Appendix S1.

#### Comparison between the selected piecewise constant model and the selected piecewise linear model

**Figure 2:**
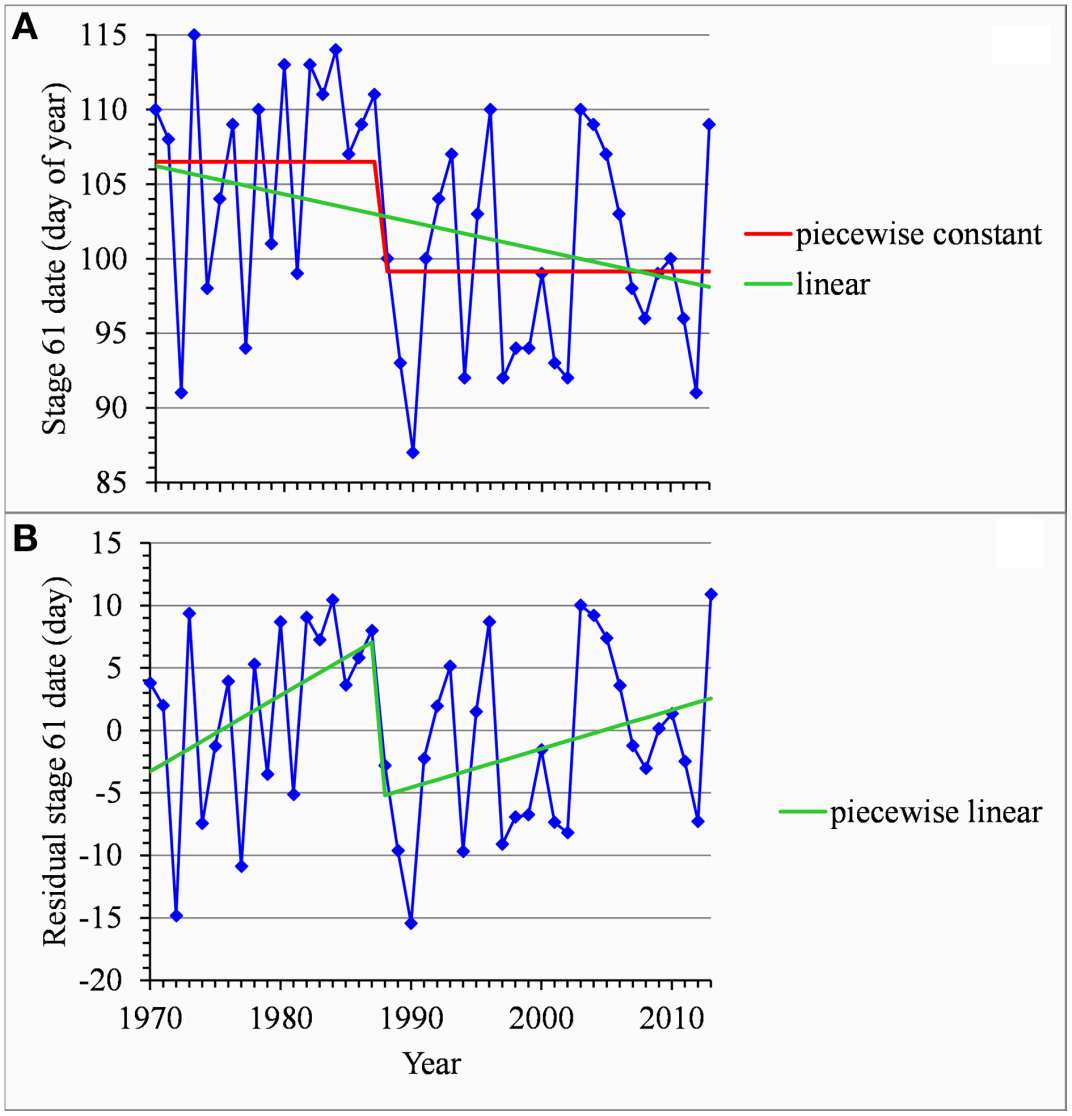
**(A)** Segmentation of the Forli BBCH 61 stage date series using a 2-segment piecewise constant model and estimation of a linear model. **(B)** Segmentation of the residual series deduced from the estimated linear model using a 2-segment piecewise linear model.

**Figure 3:**
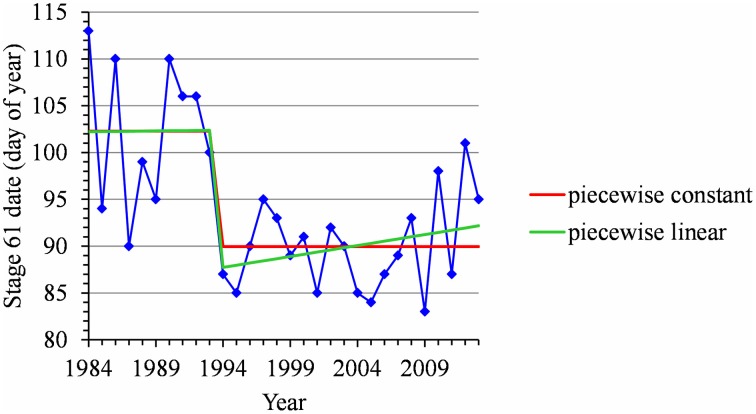
**Segmentation of the Ain Taoujdate BBCH 61 stage date series using a 2-segment piecewise constant model and a 2-segment piecewise linear model**.

**Figure 4:**
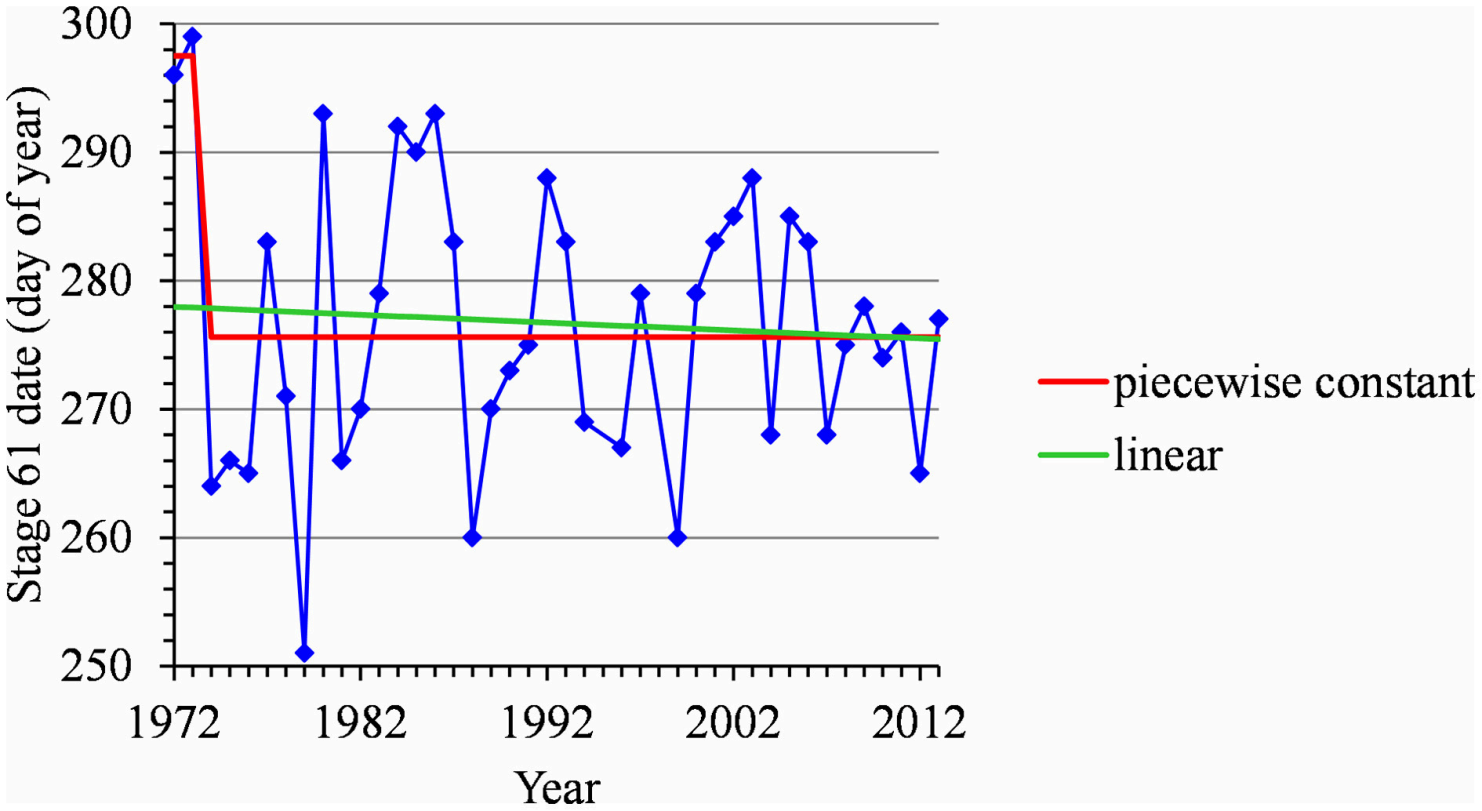
**Segmentation of the Sao Joaquim Golden Delicious BBCH 61 stage date series using a 2-segment piecewise constant model and estimation of a linear model**.

For many flowering date series, we obtained two models that were not discernible according to mBIC: the 2-segment piecewise constant model and the simple linear model (i.e., 1-segment piecewise linear model). This situation is illustrated by the Forli series (Figure 2A) for which the difference of mBIC is < 1. This can be explained by the similar orders of magnitude for the change-point amplitude and the global standard deviation in the case of the 2-segment piecewise constant model. We thus extracted the residual series from the linear function and we found that the residual series was not stationary but that a change point can be identified in 1988 (this was the change point of the selected 2-segment piecewise constant model), between two increasing linear trends (for this, we selected the best piecewise linear model for the residual series using mBIC; Figure 2B). The Ain Taoujdate series illustrates another situation where the 2-segment piecewise constant model can be identified using piecewise linear models (the selected model in this family was a 2-segment model and the two estimated slopes were not significantly different from 0; Figure 3). Finally, the Sao Joaquim series for Golden Delicious illustrates the case of very short segments at one end of the series (Figure 4). In this situation, we chose to not consider these very short segments that cannot be interpreted in our application context.

#### Assessment of the segmentation assumption

It is often of interest to quantify the uncertainty concerning change point instants. Let $L_{J}\left(\text{s},\text{x};\hat{\theta }\right)$ denote the likelihood of the segmentation **s** in *J* segments of the observed series **x** where θ denotes the set of within-segment and global variance parameters. In the case of a single change point (*J* = 2), the posterior probability of entering the second segment at τ_1_ is given by:

(2) $$L_{2}(s(\tau_{1}),x;\hat{\theta })/\sum_{s}L_{2}(s,x;\hat{\theta }),$$

where each segmentation **s** defines a unique change point. In our case of short series, the dynamic programming algorithm for computing the top *N* most probable segmentations proposed by Guédon (2013) was used to compute the *T* − 1 possible segmentations and the associated likelihood and then to extract the change-point posterior distribution. In this particular case of a single change point, this posterior distribution therefore summarizes the possible segmentations. In particular, the posterior probability of the optimal segmentation s^*^ given by:

(3) $$P(s^{*}|x;2)=L_{2}(s^{*},x;\hat{\theta })/\sum_{s}L_{2}(s,x;\hat{\theta }),$$

which is the mode of the change-point posterior distribution, can be used to assess the segmentation assumption.

More generally, the posterior probability of the optimal segmentation given by:

(4) $$P(s^{*}|x;J)=L_{J}(s^{*},x;\hat{\theta })/\sum_{s}L_{J}(s,x;\hat{\theta }),$$

can be computed using the dynamic programming algorithm for computing the top *N* most probable segmentations in our case of short series segmented into a few segments (up to *J* = 3). The assessment of multiple change-point models thus relies on two posterior probabilities:

- Posterior probability of the optimal segmentation s^*^ for a fixed number of segments *J P*(s^*^|**x**; *J*) i.e., weight of the optimal segmentation among all the possible segmentations for a fixed number of segments.
- Posterior probability of the *J*-segment model *M*_*J*_, *P*(*M*_*J*_|***x***) deduced from the mBIC computed for a collection of multiple change-point models for *J* = 1, …, *J*_max_ i.e., weight of the *J*-segment model among all the possible models between 1 and *J*_max_ segments; see Supplementary Material, Appendix S1.

## Results

In this study, we systematically favored longitudinal analyses of the various series (flowering dates and durations, mean temperatures during the CA and HA periods) in order to identify phenological patterns with minimum a priori assumptions. We also chose to not build simple regression models on the basis of these longitudinal data since this would rely on an oversimplified view of the influence of the temperatures on the flowering process regarding the biological bases and current functional models of bud phenology (Cook et al., 2012; Guo et al., 2013; Darbyshire et al., 2014; Pope et al., 2014).

### Flowering time

The dates of stages 61 and 65 appeared highly correlated, meaning that flowering durations fluctuate around quite constant values for a given series and that the changes in dates of stages 61 and 65 are markedly synchronous for a given series (see Section Flowering Duration). We thus focused the analysis on the 15 series of stage 61 dates for which data were collected at nine locations. We also analyzed the 2 series of stage 65 dates in Conthey for which only stage 65 dates were collected over long periods (44 and 39 years for Golden Delicious and Gala, respectively; Table 1).

Combining model selection criterion (mBIC) and residual analysis in the case of piecewise linear models, we found that the assumption of a piecewise constant model was better supported than the assumption of a piecewise linear model. In order to ease comparison between locations and cultivars, we chose to focus on 2-segment piecewise constant models. This corresponds to the models selected by mBIC for seven flowering date series: Angers (Golden Delicious), Forli (Golden Delicious), Trento (Golden Delicious), Gembloux (Golden Delicious), Conthey (Golden Delicious and Gala), and Ain Taoujdate (Golden Delicious). This was a well-supported alternative model for four other flowering date series: Nîmes (Gala and Fuji), Caçador (Golden Delicious and Gala) according to the posterior probability of the 2-segment model (Table 2). It should be noted that in our context of short series (length between 25 and 56), the number of segments given by mBIC should only be considered as indicative. We chose to discard 2-segment piecewise constant models selected by mBIC for Sao Joaquim (Golden Delicious—Figure 4—and Gala) since this corresponds to very short segments at the beginning of the series (2 and 1 years respectively) that cannot be reliably interpreted in our context (Table 3). Two-segment piecewise constant models are well-defined if the single change point of sufficient amplitude with respect to the global segment standard deviation separates two sufficiently long segments. It should be noted that the 3-segment model selected by mBIC for Bonn includes a short 4-year segment (between 1958 and 1961) at the beginning of the series. Since this range of years was not represented in other series, it was difficult to interpret this first segment. The 2-segment model retained for comparison of locations (Table 2) was simply this optimal 3-segment model where the first two segments were merged (Table 3; Figure S1 in Supplementary Material). No change point can be detected for Sao Joaquim (Fuji) and Caçador (Fuji). In the case of 2-segment models the change point is located between 1987 and 1989 for most of the flowering date series, which is consistent with our previous analyzes (Guédon and Legave, 2008), but with the notable exceptions of Ain Taoujdate (change point in 1994; Table 2). For the flowering series starting at the beginning of the 1980s with a change point detected at the end of the 1980s, Nîmes (Fuji), Trento (Golden Delicious), Gembloux (Golden Delicious), Caçador (Golden Delicious, Gala), the rather short length of the first segment (between 3 and 6 years) makes the mean estimated for this segment less reliable and, consequently, the change-point amplitude. This explains the difference in change-point amplitude for Nîmes between Fuji and the other two cultivars, as well as the difference between Gembloux (Golden Delicious) and Bonn (Golden Delicious; Table 2) for which the climatic conditions were rather similar (Table 1).

**Table 2:** **Segmentations of flowering date series (BBCH 61 stage for all locations except Conthey—BBCH 65 stage) using piecewise constant models (2 or 1 segment when the 2-segment model was irrelevant): observation period, change-point instant and amplitude, global standard deviation, optimal segmentation posterior probability, model posterior probability, mBIC model, average flowering duration, correlation coefficient between BBCH 61 and 65 stage dates**.

**Location**	**Cultivar**	**Observation period**	**Change point**		**Standard deviation**	**Posterior probability**		**mBIC model**	**Average flowering duration**	**61–65 stage date correlation coef**.
			**Instant**	**Amplitude**		**Segmentation**	**Model**			
Angers	Golden D.	1963–2013	1989	−7.81	6.89	0.33	0.81^*^			
Nîmes	Golden D.	1974–2013	1989	−5.39	7.05	0.15	0.1	1	2.71	0.99
Nîmes	Gala	1979–2013	1989	−6.7	6.59	0.25	0.2	1	2.68	0.99
Nîmes	Fuji	1983–2013	1988	−11.2	7.51	0.24	0.27	1	3.15	0.96
Forli	Golden D.	1970–2013	1988	−7.35	6.87	0.36	0.62^*^		3.95	0.97
Trento	Golden D.	1983–2013	1988	−13.75	5.96	0.32	0.52^*^		3.58	0.98
Gembloux	Golden D.	1984–2013	1987	−15.44	5.63	0.64	0.92^*^			
Bonn	Golden D.	1958–2013	1989	−9.33	7.96	0.33	0.12	3	4.45	0.95
Conthey (65)	Golden D.	1970–2013	1988	−8.3	5.45	0.43	0.78^*^			
Conthey (65)	Gala	1975–2013	1988	−9.88	5.25	0.52	0.71^*^			
Ain Taoujdate	Golden D.	1984–2013	1994	−12.35	5.97	0.73	0.73^*^		14.3	0.87
Sao Joaquim	Golden D.	1972–2013	–	–	11.05	1	0.37	2	9.33	0.92
Sao Joaquim	Gala	1972–2013	–	–	9.95	1	0.21	2	10.24	0.89
Sao Joaquim	Fuji	1976–2013	–	–	10.33	1	0.98^*^		7.92	0.95
Caçador	Golden D.	1984–2013	1988	−13.29	9.49	0.19	0.27	1	10.96	0.91
Caçador	Gala	1982–2013	1988	−14.75	8.8	0.47	0.33	3	10.78	0.91
Caçador	Fuji	1982–2013	–	–	11.2	1	0.52^*^		13.53	0.78

*In the model posterior probability column, an asterisk indicates that the model is the one given by mBIC. If this is not the case, the model given by mBIC is indicated in the next column (mBIC model)*.

**Table 3:** **Optimal segmentations of flowering date series (BBCH 61 stage) using piecewise constant models for series where the optimal ***J***-segment model according to the mBIC was not retained for the comparison in Table 2: observation period, change-point instant and amplitude, global standard deviation, optimal segmentation posterior probability, model posterior probability**.

**Location**	**Cultivar**	**Observation period**	**Change point 1**		**Change point 2**		**Standard deviation**	**Posterior probability**	
			**Instant**	**Amplitude**	**Instant**	**Amplitude**		**Segmentation**	**Model**
Nîmes	Golden D.	1974–2013	–	–			7.44	1	0.87
Nîmes	Gala	1979–2013	–	–			7.12	1	0.77
Nîmes	Fuji	1983–2013	–	–			8.17	1	0.68
Bonn	Golden D.	1958–2013	1962	13.81	1989	−11.12	7.21	0.28	0.49
Sao Joaquim	Golden D.	1972–2013	1974	−21.87			10.06	0.58	0.47
Sao Joaquim	Gala	1972–2013	1975	−29.16			8.87	0.72	0.73
Caçador	Golden D.	1984–2013	–	–			10.53	1	0.54
Caçador	Gala	1982–2013	1988	−13.42	2012	−17.25	7.82	0.27	0.56

**Figure 5:**
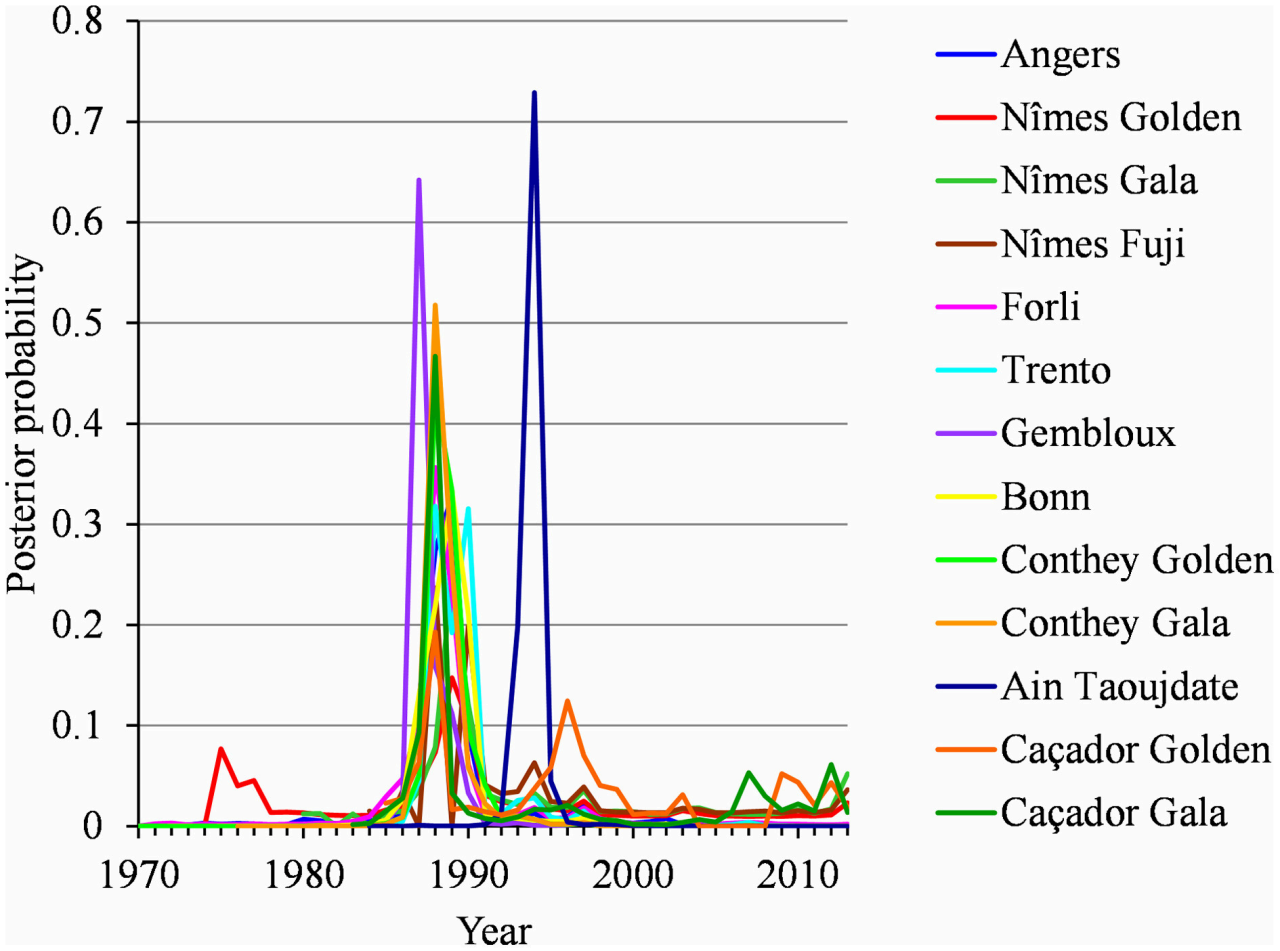
**Two-segment piecewise constant models estimated on the basis of BBCH 61 stage date series for Angers (Golden Delicious), Nîmes (Golden Delicious, Gala, and Fuji), Forli (Golden Delicious), Trento (Golden Delicious), Gembloux (Golden Delicious), Bonn (Golden Delicious), Ain Taoujdate (Golden Delicious), and Caçador (Golden Delicious and Gala) and BBCH 65 stage date series for Conthey (Golden Delicious and Gala): posterior change-point distributions**.

**Figure 6:**
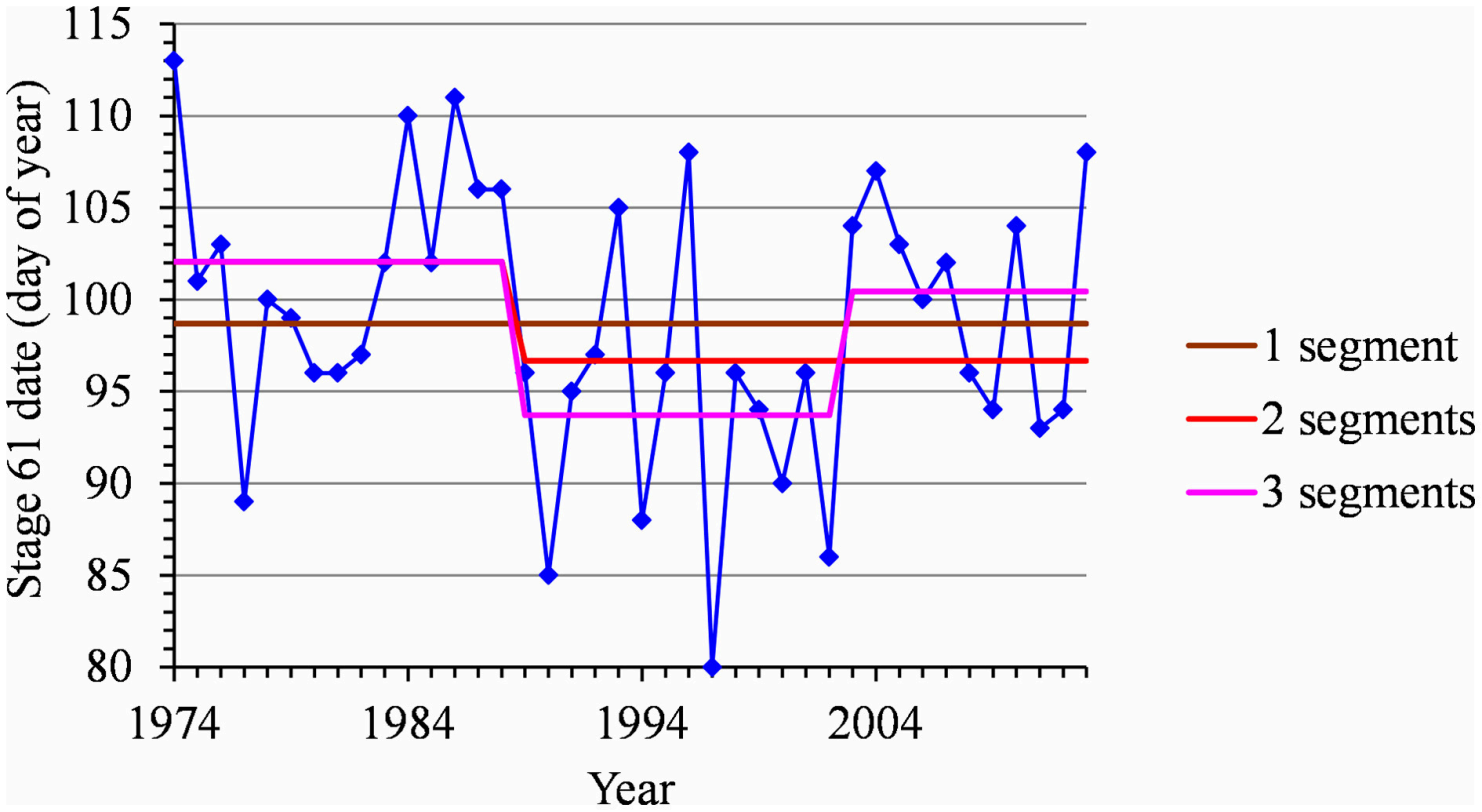
**Segmentation of the Nîmes Golden Delicious BBCH 61 stage date series using 1-, 2-, and 3-segment piecewise constant models**.

For each flowering date series, the uncertainty concerning the instant of the change point is low for most locations (Figure 5) except for Nîmes (Golden Delicious) for which the posterior probability of the segmentation is the lowest among the segmentation in 2-segments (Table 2). Moreover, this series is the only one for which the change-point amplitude is lower than the global standard deviation in the case of a 2-segment piecewise constant model (Table 2). Hence, the segmentation in 2-segments is not well defined in this case. This can be illustrated by the segmentation in 2- and 3-segments of this flowering date series where the segmentation in 3-segments highlights a change toward later flowering dates since 2003 (Figure 6). It should be noted that in the case of Nîmes, the mBIC favors the constant model (i.e., no change point) regardless of the cultivar (Tables 2, 3).

### Flowering duration

**Figure 7:**
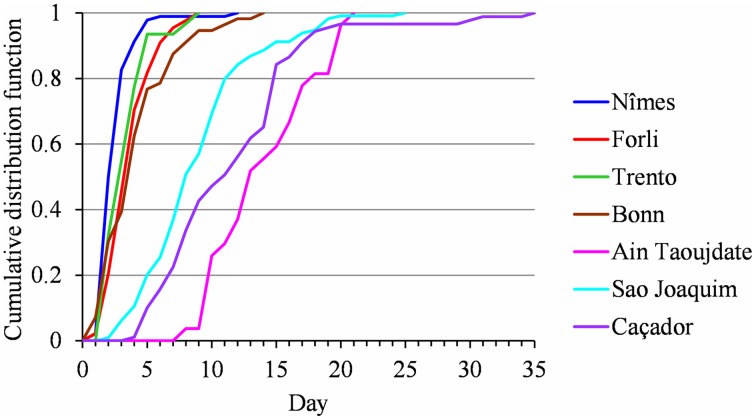
**Flowering duration for Nîmes (Golden Delicious, Gala, and Fuji samples pooled), Forli, Trento, Bonn, Ain Taoujdate; Sao Joaquim (Golden Delicious, Gala, and Fuji samples pooled) and Caçador (Golden Delicious, Gala, and Fuji samples pooled): comparison of cumulative frequency distribution functions**.

We did not detect any change point or linear trend in the flowering duration series and we thus analyzed them as simple frequency distributions without considering the year indexing (Table 2). We first grouped samples corresponding to the different cultivars in a given location (Golden Delicious, Gala and Fuji for Nîmes, Sao Joaquim and Caçador, respectively) for which the frequency distributions were not significantly different according to the Kruskal Wallis test (ANOVA by ranks for these frequency distributions defined on a small set of values). The cumulative frequency distribution functions of the seven samples (Figure 7) highlights a clear order for flowering duration (from the shortest to the longest): (1) Nîmes, 2.82 days on average; (2) Forli, Trento and Bonn, between 3.6 and 4.45 days (these three samples are not significantly different according to the Kruskal Wallis test); (3) Sao Joaquim, 9.18 days; (4) Caçador, 11.82 days; (5) Ain Taoujdate, 14.3 days.

### Temperature during the CA and HA periods vs. flowering date and duration

**Figure 8:**
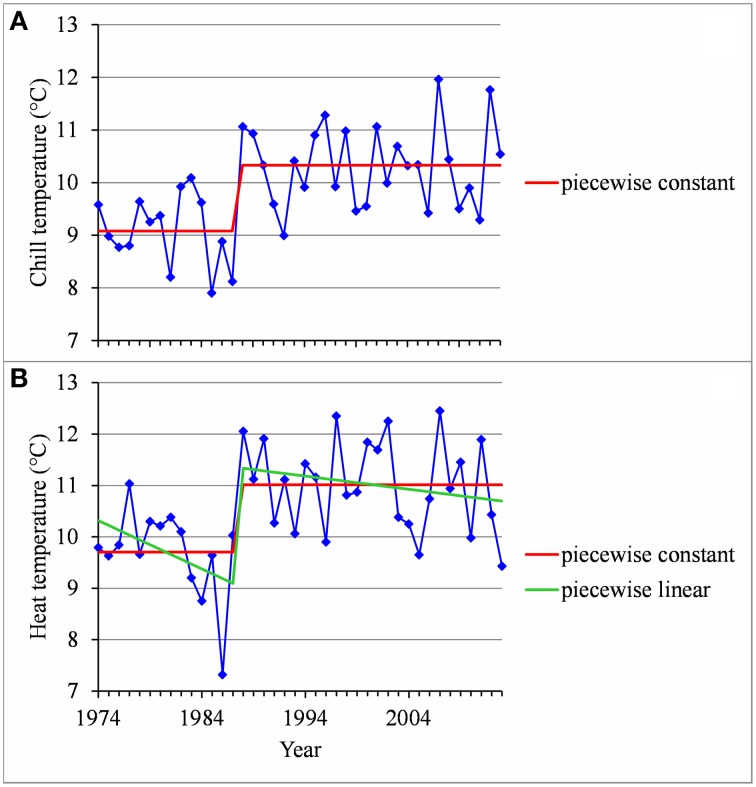
**(A)** Segmentation of the Nîmes series of mean temperatures during the CA period using a 2-segment piecewise constant model. **(B)** Segmentation of the Nîmes HA period temperature series using a 2-segment piecewise constant model and a 2-segment piecewise linear model.

The flowering date was not significantly correlated with the mean temperature during the CA period (defined for the Northern Hemisphere) for most of the European locations (Table 4). The only exceptions were Trento and Conthey for which we found slightly significant negative correlations. The situation was very different for the Brazilian locations for which we found strongly significant positive correlations (except for Caçador, cultivar Fuji) between the flowering date and the mean temperature during the CA period (defined for the Southern Hemisphere), which means that the warmer the austral CA period is, the later the flowering date will be. The Moroccan situation seems to be closer to the Brazilian situations than to the European ones but we cannot be conclusive in this case because the correlation coefficient was not significantly different from 0 (Table 4). We thus conducted a longitudinal analysis of the series of mean temperatures during the CA period using the methodology previously applied to the flowering date series. We found various patterns:

- Two stationary segments for Nîmes (1974–2013, change point in 1988; Figure 8A) and Forli (1970–2013, change point in 1993), but with a rather small change-point amplitude with respect to the residual standard deviation in this latter case.
- Stationary series for Trento (1983–2013), Ain Taoujdate (1973–2013) and Sao Joaquim (1955–2013).
- Slightly positive slope for Angers (1963–2013), Gembloux (1964–2013), Bonn (1959–2013), Conthey (1970–2013) and Caçador (1961–2013).

**Table 4:** Correlation coefficients between flowering date (BBCH 61 stage for all locations except Conthey—BBCH 65 stage) and mean temperature during the CA period (^**^significant at 1% level; ^*^significant at 5% level; n.s., non-significant).

**Location**	**Observation Period**	**Correlation coefficient**		
		**Golden D**.	**Gala**	**Fuji**
Angers	1963–2013	−0.2 n.s.		
Nîmes	1974–2013	0 n.s.	−0.05 n.s.	−0.05 n.s.
Forli	1970–2013	−0.05 n.s.		
Trento	1983–2013	−0.3 n.s.		
Gembloux	1984–2013	−0.19 n.s.		
Bonn	1959–2013	−0.22 n.s.		
Conthey	1970–2013	−0.4^**^	−0.36^*^	
Ain Taoujdate	1984–2013	0.19 n.s.		
Sao Joaquim	1972–2013	0.69^**^	0.63^**^	0.72^**^
Caçador	1982–2013	0.63^**^	0.61^**^	0.25 n.s.

**Figure 9:**
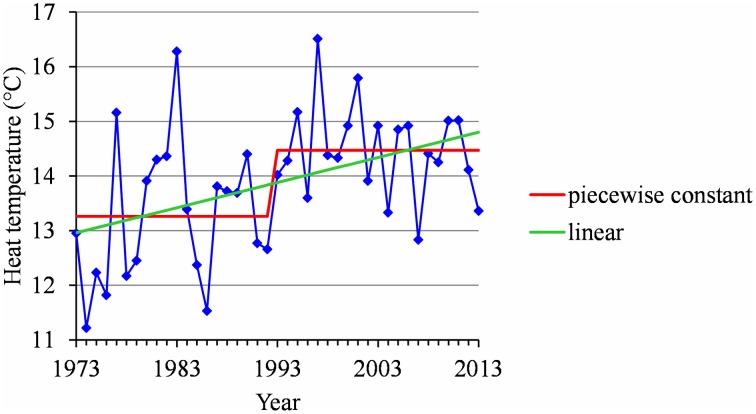
**Segmentation of the Ain Taoujdate series of mean temperatures during the HA period using a 2-segment piecewise constant model and estimation of a linear model**.

We found strongly significant negative correlations for the European locations between the flowering date and the mean temperature during the HA period (defined for Europe), which means that the warmer the Northern HA period is, the earlier the flowering date will be (Table 5). The situation was very different for the Brazilian locations for which the flowering date was not significantly correlated with the mean temperature during the HA period defined in Brazil (Table 5). The only exception was Sao Joaquim, cultivar Golden Delicious, for which we found a slightly significant negative correlation. For the Moroccan location we found a significant negative correlation between the flowering date and the mean temperature during the HA period. This relationship appears to be closer to the relationships found for the European locations than to the ones found for the Brazilian locations (Table 5). Because of the significant correlations between the flowering date and the mean temperature during the HA period found for the European and Moroccan locations, we conducted a longitudinal analysis of the series of mean temperatures during this period (Table 5) using the methodology previously applied to the flowering date series. We found a change point at the end of the 1980s in all the European temperature series when applying the piecewise constant model (see an illustration in Figure 8B for Nîmes) and the change-point amplitude was around 1.3°C for most locations (it should be noted that for Gembloux, the change-point amplitude estimated on the complete series up to 1966 is far more reliable than the one estimated on the series corresponding to the flowering date range of years since, in this latter case, the first segment was very short—four years). We found a change point of similar amplitude but in 1993 in the Moroccan location when applying a piecewise constant model (Table 5), although the assumption of a simple linear model was better supported (Figure 9). In contrast, the series of mean temperatures during the HA period were stationary for the Brazilian locations (Table 5).

**Table 5:** Segmentations of series of mean temperatures during the HA period using piecewise constant models (2 or 1 segment when the 2-segment model was irrelevant): recording period, change-point instant and amplitude, global standard deviation, optimal segmentation posterior probability, model posterior probability, mBIC model, correlation coefficients between flowering date (BBCH 61 stage for all locations except Conthey–BBCH 65 stage) and mean temperature during the HA period (^**^significant at 1% level; ^*^significant at 5% level; n.s., non-significant).

**Location**	**Recording period**	**Change point**		**Standard deviation**	**Posterior probability**		**mBIC model**	**Correlation coefficient**		
		**Instant**	**Amplitude**		**Segmentation**	**Model**		**Golden D**.	**Gala**	**Fuji**
Angers	1963–2013	1987	1.35	0.94	0.63	0.16	3	−0.77^**^		
Nîmes	1974–2013	1988	1.31	0.88	0.57	0.37	3	−0.7^**^	−0.72^**^	−0.76^**^
	1966–2013	1988	1.26	0.9	0.56	0.45^*^				
Forli	1970–2013	1988	0.91	0.92	0.26	0.42	1	−0.8^**^		
Trento	1983–2013	1988	1.72	0.89	0.54	0.81^*^		−0.78^**^		
Gembloux	1984–2013	1988	2.84	1.19	0.48	0.46^*^		−0.81^**^		
^†^	1964–2013	1989	1.49	1.16	0.29	0.57^*^				
Bonn	1959–2013	1989	1.34	1.26	0.27	0.59^*^		−0.84^**^		
Conthey	1970–2013	1988	1.31	1.06	0.31	0.7^*^		−0.78^**^	−0.74^**^	
Ain Taoujdate	1984–2013	1993	1.32	0.86	0.33	0.75^*^		−0.47^**^		
	1973–2013	1993	1.21	1.08	0.14	0.52^*^				
Sao Joaquim	1972–2013	-	-	0.76	1	0.91^*^		−0.34^*^	−0.27 n.s.	−0.12 n.s.
	1955–2013	-	-	0.79	1	0.97^*^				
Caçador	1982–2013	-	-	0.69	1	0.74^*^		−0.23 n.s.	−0.2 n.s.	−0.05 n.s.
	1961–2013	-	-	0.8	1	0.76^*^				

*In the model posterior probability column, an asterisk indicates that the model is the one given by mBIC. If this is not the case, the model given by mBIC is indicated in the next column (mBIC model). We analyzed the mean temperatures during the HA period over the range of years corresponding to the flowering dates and in certain cases (Nîmes, Gembloux, Ain Taoujdate, Sao Joaquim, Caçador) over an extended range of years*.

† *Gembloux (1964–2013): Posterior probability of 0.29 for 1989 but of 0.27 for 1988*.

We also found non-significant correlations between the flowering durations and the mean temperatures, both during the CA and HA periods at all the seven locations where these correlations were analyzed (especially in Morocco and Brazil; results not shown).

## Discussion

### Differentiated flowering date series

The analyses showed clear advances of apple flowering time since the end of the 1980s in most locations of Western Europe and a few years later in Northern Morocco, whereas the flowering dates remained stationary in Southern Brazil. Our study thus provides a new overview in space and time of the flowering time changes in apple tree highlighting contrasting behaviors (advance or stationarity) contrary to previous studies that only reported flowering advances (Chmielewski et al., 2004; Wolfe et al., 2005; Guédon and Legave, 2008; Eccel et al., 2009; Fujisawa and Kobayashi, 2010; Grab and Craparo, 2011; Darbyshire et al., 2013). Nevertheless, the flowering advances have been found to a lesser amplitude in the Southern Hemisphere (Australia, Southern Africa) than in the Northern Hemisphere (Europe, Japan, United States) as outlined by Darbyshire et al. (2013). Such lesser changes in flowering date in the Southern Hemisphere are consistent with the stationary series mainly found in Southern Brazil.

While there was a strong consensus concerning abrupt flowering advances in Europe, a notable exception was the French Mediterranean location (Nîmes) for which the flowering dates were stationary. This was clearly demonstrated for Golden Delicious for which the flowering advance reported by Guédon and Legave (2008) up to 2002 became an alternative not strongly supported when records up to 2013 were included in the series.

When all our findings are taken into account, the overview of flowering date changes reveals that flowering advances or stationary flowering dates may be detected in temperate as well as in mild regions: (i) advances for most European series, the Moroccan series and one Brazilian series; and (ii) stationarity for the French Mediterranean series and most Brazilian series. A simple relationship between the flowering date change pattern and a geographical trait such as the elevation or the localization in the Northern or Southern Hemisphere is no longer valid.

### Relationships between temperatures and flowering dates

The correlation analysis gave evidence of contrasting climatic determinisms of flowering time in relation to the localization in either temperate or mild regions. In Western Europe, our analysis showed that the higher the HA is the earlier the flowering date will be, whereas the CA was not related to the flowering time. These results emphasize that flowering time in recent past have been strongly determined by the HA in the temperate conditions of Western Europe. This was similarly showed for apple tree in other temperate conditions like those of Northeastern America and Japan (Wolfe et al., 2005; Fujisawa and Kobayashi, 2010).

In Southern Brazil, a main influence of CA on flowering time was emphasized since the correlation analysis mainly showed that the lower the CA is the later the flowering date will be, as would be expected in mild conditions (Atkinson et al., 2013). Although a similar determinism of flowering time was highlighted in Northern Morocco (Oukabli et al., 2003), this appeared less evident on the basis of our correlation analysis. That may be explained by an additional influence of the HA, which was shown by the correlation analysis in Northern Morocco. Nevertheless, our results globally showed that flowering time would be mainly determined by the CA in mild conditions of both Southern Brazil and Northern Morocco. Such determinism is consistent with the high genetic heritabilities of the chill requirement trait that were found from apple progenies grown in mild conditions like those of Southern Africa (Labuschagné et al., 2002). In addition severe symptoms of inadequate chill requirements, including delays of floral budbreak and flowering time were observed in high-chill genotypes selected from Golden Delicious progenies. Therefore, it was generally accepted in apple tree that budbreak time in mild regions was an accurate biomarker of the fulfillment of chill requirements (Hauagge and Cummins, 1991b). This supposes a short time between the fulfillment of chill requirements and floral budbreak in mild conditions, which was demonstrated in Southern Brazil by comparison with the temperate conditions of Southern France (Malagi et al., 2015).

The analysis of temperature series accounts for contrasting warming contexts in relation to the localization in the Northern or Southern Hemisphere. In Western Europe, a marked warming during the HA period was detected at the end of the 1980s, similarly to the change-point instant generally found for the flowering date. Such a concomitance was also detected in Northern Morocco at the beginning of the 1990s. Heat increase can thus explain flowering advances in Western Europe where the HA would mainly determine flowering time and in Northern Morocco where the influence of HA would be also involved. In Southern Brazil, the stationarity of temperatures during both the CA and HA periods can explain the stationarity of most flowering date series. The absence of warming in Southern Brazil over the last four decades could be attributed to the relatively high elevation of the studied locations and to the globally lower temperature increases since the 1970s in the Southern Hemisphere compared to the Northern Hemisphere (Jones and Moberg, 2003).

As a consequence of these relationships, flowering date advances and stationary flowering dates result from interactions between contrasting thermal determinisms (temperate vs. mild regions) and warming contexts (Northern vs. Southern Hemisphere). A particularly interesting result is the stationarity of flowering dates both in the Brazilian and the French Mediterranean regions. In Southern Brazil no thermal influences could have been exercised in the absence of warming, whereas the French Mediterranean region would have been progressively submitted to two opposite thermal influences: an increase in HA compensated by a decrease in CA, respectively linked to marked warming during the HA and CA periods. While less marked, the decrease of CA tended to delay the flowering dates. This appears mainly due to certain years since 1988, including 1988, 1996, 2007, and 2012, that were characterized by relatively high temperatures during the CA period (Figure 8A). This may explain the absence of correlation up to 2013 between the flowering date and the temperature during the CA period in this Mediterranean region.

Stationary flowering dates resulting from two opposite warming impacts, such as in the French Mediterranean region, may thus cast doubt on the relevance of flowering advances in apple tree as a suitable indicator of recent warming in early spring in Europe (Menzel et al., 2011). This indicator would be even more questionable in the near future in the entire Mediterranean region, as suggested by a similar temperature increase during the CA period since 1993 in the Italian Mediterranean region of Forli near the Adriatic coastline (see Section Temperature During the CA and HA Periods vs. Flowering Date and Duration).

There are still many unknowns concerning the relationships between the flowering dates and temperatures from bud dormancy to flowering time. This requires new researches on the timings of fulfillment of the chill and heat requirements and better knowledge of temperature thresholds on flowering time (Guo et al., 2013; Luedeling et al., 2013; Pope et al., 2014).

### Specificity of the flowering duration

As expected (Atkinson et al., 2013), our analyses highlighted contrasting flowering duration patterns between temperate (short durations) and mild conditions (far longer durations). An additional specificity of flowering duration patterns is the lack of detection of a change point or linear trend at all locations, contrary to the flowering time patterns. This suggests different climatic determinisms for the flowering duration and the flowering time. In temperate conditions, a likely climatic determinism of flowering duration is the HA rate during the blooming phase, which is consistent with a significant increase in the average flowering duration from the Mediterranean location of Nîmes (2.8 days) to the colder continental location of Bonn (4.5 days). In mild conditions inversely, longer durations of the blooming phase are currently attributed to insufficient CAs (Campoy et al., 2011). Nevertheless, our results suggest more complex thermal determinisms to explain extended flowering durations in mild conditions since non-significant correlations were found between the flowering duration and the mean temperature during the CA period as well as during the HA period in both the Moroccan and Brazilian locations (see Section Temperature During the CA and HA Periods vs. Flowering Date and Duration).

### Strengths of the statistical modeling approach

Homoscedastic 2-segment piecewise constant models, where the variance was assumed to be common to the two segments, played a key role in this study. This homoscedasticity assumption can be assessed by examining the standard deviations empirically estimated for the two segments in the case of sufficiently long flowering date or series of mean temperatures during the HA period (see Supplementary Materials, Tables S1, S2). In our context of potentially short segments, homoscedastic models are more robust than heteroscedastic models where changes in variance can induce artifactual short segments. One shortcoming of homoscedastic models with respect to heteroscedastic models used in our previous study (Guédon and Legave, 2008) was the inability to compute posterior change-point distributions. This is no longer the case since, for a single change point, the posterior change-point distribution can be computed using the dynamic programming algorithm for computing the top *N* most probable segmentations proposed by Guédon (2013). More generally, the posterior probability of the optimal segmentation can be computed using this dynamic programming algorithm in our case of short series segmented in a few segments. The other main methodological improvement consists in the systematic comparison of homoscedastic piecewise constant models with homoscedastic piecewise linear models. The fact that simple linear models corresponding to the linear trend assumption were systematically compared with piecewise constant models and the ability to identify piecewise constant models using piecewise linear models (see the Ain Taoujdate example in Figure 3) strengthen the reliability of our results with respect to our previous study.

### Implications of flowering changes on climatic adaptation

Early flowering dates might increase the risk of spring frost damages, as pointed out for a long time by Cannell and Smith (1986) in Great Britain. More recently, spring frosts in southern regions of Great Britain have been seen to be decreasing both in frequency and severity (Sunley et al., 2006). Likewise, investigations into the frost risk for apple tree in Northern Italy (Eccel et al., 2009) showed that the risk was lower than in the past, and suggested that it will remain stable or decrease slightly in the future. Thus, the frost risk during the fruit tree flowering phase in Europe will probably be more open to debate in the future context of climate warming because of regional differences in both the magnitude of flowering advances and the frequency of negative temperatures. In particular, our results suggest that the French Mediterranean region may be rarely subject to frost risk at present time because flowering advance is decreasing. Inversely, this risk might remain a true concern for growers in continental regions because of constant flowering advances and also relatively short flowering durations (as shown in Bonn in Germany).

Mediterranean and oceanic regions in Europe might be more affected in the future by excessive delays in chill fulfillment, particularly the French Mediterranean region where our study showed an increase in temperature from October to January since the end of the 1980s. Declining chill will become a limiting cropping factor (Atkinson et al., 2013) and a new warming vulnerability in many European Mediterranean regions, particularly for the apple tree characterized by high chill requirements for most commercial cultivars. At a certain point, CA might shift from sub-optimal, just inducing a delayed phenology as illustrated in our study by the Nîmes location, to below the requirement leading to irregular or insufficient yields. At the orchard scale, this could cause in the future phenological disorders similar to those observed in the Mediterranean mild conditions of Northern Morocco (Oukabli et al., 2003), such as insufficient flowering synchronization between non self-fertile cultivars (all apple cultivars), which require cross-pollination, and excessive duration of the fruit maturity phase, that may be consequences of late and extended flowering.

## Conclusion

The collection of flowering date series recorded in contrasting climatic locations and their analysis based on multiple change-point models proved to be appropriate for identifying differentiated flowering patterns in apple tree. The same methodology applied to the corresponding temperature series provided complementary results, making possible to establish a comprehensive overview of the relationships between the flowering phenology and the warming context at the world scale. On the one hand, we showed that the different patterns of flowering time change are not closely related to the localization of apple trees in temperate or mild regions or in the Northern or Southern Hemisphere. On the other hand, contrasting temperature influences during successive CA and HA periods on flowering time appeared to be mainly in relation with the localization in temperate or mild conditions, while contrasting warming contexts appeared to be in relation with the localization in the Northern or Southern Hemisphere. This overview was completed by information on the relationships between flowering duration and temperature, which appeared to be different and more complex than those concerning the flowering time. Because continuous warming will change the relationships between phenology and temperature, a new warming vulnerability is expected in the more or less long term in Europe especially in Mediterranean regions where apple tree is a native crop.

## Author contributions

JL conceived the study, conducted the data collection, contributed to the data analysis and edited the manuscript; YG contributed to the study conception, conducted the statistical analysis and edited the manuscript; GM and AE supplied data for Brazil and Morocco respectively, contributed to the data analysis and manuscript approval; MB contributed to the study conception, the data analysis, and manuscript approval.

### Conflict of interest statement

The authors declare that the research was conducted in the absence of any commercial or financial relationships that could be construed as a potential conflict of interest.

## References

[B1] Abu-asab M. S. Peterson P. M. Shetler S. G. Orli S. S. (2001). Earlier plant flowering in spring as a response to global warming in the Washington, DC, area. Biodivers. Conserv. 10, 597–612. 10.1023/A:1016667125469

[B2] Atkinson C. J. Brennan R. M. Jones H. G. (2013). Declining chilling and its impact on temperate perennial crops. Env. Exp. Bot. 91, 48–62. 10.1016/j.envexpbot.2013.02.004

[B3] Auger I. E. Lawrence C. E. (1989). Algorithms for the optimal identification of segment neighborhoods. Bull. Math. Biol. 51, 39–54. 10.1007/BF02458835 2706400

[B4] Campoy J. A. Ruiz D. Egea J. (2011). Dormancy in temperate fruit trees in a global warming context: a review. Sci. Hortic. 130, 357–372. 10.1016/j.scienta.2011.07.011

[B5] Cannell M. G. R. Smith R. I. (1986). Climatic warming, spring budburst and frost damage on trees. J. Appl. Ecol. 23, 177–191. 10.2307/2403090

[B6] Chmielewski F. M. Müller A. Bruns E. (2004). Climate changes and trends in phenology of fruit trees and field crops in Germany, 1961–2000. Agric. For. Meteorol. 121, 69–78. 10.1016/S0168-1923(03)00161-8

[B7] Cook B. I. Wolkovich E. M. Parmesan C. (2012). Divergent responses to spring and winter warming drive community level flowering trends. Proc. Natl. Acad. Sci. U.S.A. 109, 9000–9005. 10.1073/pnas.1118364109 22615406 PMC3384199

[B8] Črepinšek Z. Štampar F. Kajfež-Bogataj L. Solar A. (2012). The response of *Corylus avellana* L. phenology to rising temperature in north-eastern Slovenia. Int. J. Biometeorol. 56, 681–694. 10.1007/s00484-011-0469-7 21786017

[B9] Darbyshire R. Webb L. Goodwin I. Barlow E. W. R. (2013). Evaluation of recent trends in Australian pome fruit spring phenology. Int. J. Biometeorol. 57, 409–421. 10.1007/s00484-012-0567-1 22791275

[B10] Darbyshire R. Webb L. Goodwin I. Barlow E. W. R. (2014). Challenges in predicting climate change in pome fruit phenology. Int. J. Biometeorol. 58, 1119–1133. 10.1007/s00484-013-0705-4 23877816

[B11] Doi H. Katano I. (2008). Phenological timings of leaf budburst with climate change in Japan. Agric. For. Meteorol. 148, 512–516. 10.1016/j.agrformet.2007.10.002

[B12] Eccel E. Rea R. Caffarra A. Crisci A. (2009). Risk of spring frost to apple production under future climate scenarios: the role of phenological adaptation. Int. J. Biometeorol. 53, 273–286. 10.1007/s00484-009-0213-8 19263089

[B13] El Yaacoubi A. Malagi G. Oukabli A. Hafidi M. Legave J. M. (2014). Global warming impact on floral phenology of fruit tree species in Mediterranean region. Sci. Hortic. 180, 243–253. 10.1016/j.scienta.2014.10.041

[B14] Fitchett J. M. Grab S. W. Thompson D. I. Roshan G. (2014). Increasing frost risk associated with advanced citrus flowering dates in Kerman and Shiraz, Iran: 1960-2010. Int. J. Biometeorol. 58, 1811–1815. 10.1007/s00484-013-0778-0 24429704

[B15] Fujisawa M. Kobayashi K. (2010). Apple (*Malus pumila* var. domestica) phenology is advancing due to rising air temperature in northern Japan. Glob. Chang. Biol. 16, 2651–2660. 10.1111/j.1365-2486.2009.02126.x

[B16] Garcia-Mozo H. Orlandi F. Galan C. Fornaciari M. Romano B. Ruiz L. (2009). Olive flowering phenology variation between different cultivars in Spain and Italy: modeling analysis. Theor. Appl. Climatol. 95, 385–395. 10.1007/s00704-008-0016-6

[B17] Ghariani K. Stebbins R. L. (1994). Chill requirements of apple and pear cultivars. Fruit Var. J. 48, 215–222.

[B18] Gordo O. Sanz J. J. (2009). Long-term temporal changes of plant phenology in the Western Mediterranean. Glob. Chang. Biol. 15, 1930–1948. 10.1111/j.1365-2486.2009.01851.x

[B19] Grab S. Craparo A. (2011). Advance of apple and pear tree full bloom dates in response to climate change in the southwestern Cape, South Africa: 1973–2009. Agric. For. Meteorol. 151, 406–413. 10.1016/j.agrformet.2010.11.001

[B20] Guédon Y. (2013). Exploring the latent segmentation space for the assessment of multiple change-point models. Comput. Stat. 28, 2641–2678. 10.1007/s00180-013-0422-9

[B21] Guédon Y. Legave J. M. (2008). Analyzing the time-course variation of apple and pear tree dates of flowering stages in the global warming context. Ecol. Modell. 219, 189–199. 10.1016/j.ecolmodel.2008.08.010

[B22] Guo L. Dai J. Ranjitkar S. Xu J. Luedeling E. (2013). Response of chestnut phenology in China to climate variation and change. Agric. For. Meteorol. 180, 164–172. 10.1016/j.agrformet.2013.06.004

[B23] Hauagge R. Cummins J. N. (1991a). Phenotypic variation of length of bud dormancy in apple cultivars and related Malus species. J. Am. Soc. Hortic. Sci. 116, 100–106.

[B24] Hauagge R. Cummins J. N. (1991b). Relationships among indices for the end of bud dormancy in apple cultivars and related *Malus* species. J. Am. Soc. Hortic. Sci. 116, 95–99.

[B25] Jones P. D. Moberg A. (2003). Hemispheric and large-scale surface air temperature variations: an extensive revision and an update to 2001. J. Clim. 16, 206–223. 10.1175/1520-0442(2003)016<0206:HALSSA>2.0.CO;2

[B26] Kass R. E. Raftery A. E. (1995). Bayes factors. J. Am. Stat. Assoc. 90, 773–795. 10.1080/01621459.1995.10476572

[B27] Labuschagné I. F. Louw J. H. Schmidt K. Sadie A. (2002). Genetic variation in chilling rquirement in apple progeny. J. Am. Soc. Hort. Sci. 127, 663–672.

[B28] Lang G. A. Early J. D. Martin G. C. Darnell R. L. (1987). Endo-, para-, and ecodormancy: physiological terminology and classification for dormancy research. HortScience 22, 371–377.

[B29] Legave J. M. Blanke M. Christen D. Giovannini D. Mathieu V. Oger R. (2013). A comprehensive overview of the spatial and temporal variability of apple bud dormancy release and blooming phenology in Western Europe. Int. J. Biometeorol. 57, 317–331. 10.1007/s00484-012-0551-9 22610120

[B30] Luedeling E. Kunz A. Blanke M. M. (2013). Identification of chilling and heat requirements of cherry trees - a statistical approach. Int. J. Biometeorol. 57, 679–689. 10.1007/s00484-012-0594-y 23053065 PMC3745618

[B31] Malagi G. Sachet M. R. Citadin I. Herter F. G. Bonhomme M. Regnard J. L. (2015). The comparison of dormancy dynamics in apple trees grown under temperate and mild Winter climates imposes a renewal of classical approaches. Trees 29, 1365–1380. 10.1007/s00468-015-1214-3

[B32] Menzel A. Fabian P. (1999). Growing season extended in Europe. Nature 397, 659 10.1038/17709

[B33] Menzel A. Seifert H. Estrella N. (2011). Effects of recent warm and cold spells on European plant phenology. Int. J. Biometeorol. 55, 921–932. 10.1007/s00484-011-0466-x 21755278

[B34] Miller-Rushing A. J. Katsuki T. Primack R. B. Ishii Y. Lee S. D. Higuchi H. (2007). Impact of global warming on a group of related species and their hybrids: cherry tree (Rosaceae) flowering at Mt. Takao, Japan. Am. J. Bot. 94, 1470–1478. 10.3732/ajb.94.9.1470 21636514

[B35] Muggeo V. M. R. (2003). Estimating regression models with unknown break-points. Stat. Med. 22, 3055–3071. 10.1002/sim.1545 12973787

[B36] Oukabli A. Bartolini S. Viti R. (2003). Anatomical and morphological study of apple (*Malus x domestica* Borkh) flower buds growing under inadequate winter chill. J. Hortic. Sci. Biotechnol. 78, 580–585.

[B37] Petri J. L. Leite G. B. (2004). Consequences of insufficient winter chill on apple tree bud-break. VIIIth international symposium on temperate zone fruits in the tropics and subtropics, Florianopolis (Brazil). Acta Hort. 872, 53–60. 10.17660/ActaHortic.2004.662.4

[B38] Pope K. S. Da Silva D. Brown P. H. Dejong T. M. (2014). A biologically based approach to modeling spring phenology in temperate deciduous trees. Agric. For. Meteorol. 198–199, 15–23. 10.1016/j.agrformet.2014.07.00922422014

[B39] Pope K. S. Dose V. Da Silva D. Brown P. H. Leslie C. A. Dejong T. M. (2013). Detecting nonlinear response of spring phenology to climate change by Bayesian analysis. Glob. Chang. Biol. 19, 1518–1525. 10.1111/gcb.12130 23505006

[B40] Primack R. B. Ibanez I. Higuchi H. Don Lee S. Miller-Rhushing A. J. Wilson A. M. (2009). Spatial and interspecific variability in phenological responses to warming temperatures. Biol. Conserv. 142, 2569–2577. 10.1016/j.biocon.2009.06.003

[B41] Rutishauser T. Schleip C. Sparks T. Nordli Ø. Menzel A. Wanner H. (2009). Temperature sensitivity of Swiss and British plant phenology from 1753 to 1958. Clim. Res. 39, 179–190. 10.3354/cr00810

[B42] Schwartz M. D. Hanes J. M. (2010). Continental-scale phenology: warming and chill. Int. J. Climatol. 30, 1595–1598. 10.1002/joc.2014

[B43] Sunley R. J. Atkinson C. J. Jones H. G. (2006). Chill unit models and recent changes in the occurrence of winter chill and spring frost in the United Kingdom. J. Hortic. Sci. Biotechnol. 8, 949–958. 10.1080/14620316.2006.11512181

[B44] Wolfe D. W. Schwartz M. D. Lakso A. N. Otsuki Y. Pool R. M. Shaukis N. J. (2005). Climate change and shifts in spring phenology of three horticultural woody perennials in northeastern USA. Int. J. Biometeorol. 49, 303–309. 10.1007/s00484-004-0248-9 15592880

[B45] Yu H. Luedeling E. Xu J. (2010). Winter and spring warming result in delayed spring phenology on the Tibetan Plateau. Proc. Natl. Acad. Sci. U.S.A. 107, 1–6. 10.1073/pnas.1012490107 21115833 PMC3009774

[B46] Zhang N. R. Siegmund D. O. (2007). A modified Bayes information criterion with applications to the analysis of comparative genomic hybridization data. Biometrics 63, 22–32. 10.1111/j.1541-0420.2006.00662.x 17447926

[B47] Zhang X. Tarpley D. Sullivan J. T. (2007). Diverse responses of vegetation phenology to a warming climate. Geophys. Res. Lett. 34, 1–5. 10.1029/2007gl031447

