# Supplementary material for: Differentiated Responses of Apple Tree Floral Phenology to Global Warming in Contrasting Climatic Regions
Source: Front Plant Sci. 2015 Dec 15;6:1054. doi: 10.3389/fpls.2015.01054 (PMC4678210; doi:10.3389/fpls.2015.01054)
Supplement: Supplementary file 1 [file DataSheet1.PDF]

Supplementary Material for
Differentiated responses of apple tree floral phenology to global warming in contrasting climatic regions

Jean-Michel Legave, Yann Guédon, Gustavo Malagi, Adnane El Yaacoubi, Marc Bonhomme

Appendix S1. Statistical methods for piecewise constant and piecewise linear models

Let θ denote the set of within-segment and global variance parameters. For piecewise constant models (M_{constant} models), $\theta = \{\alpha_0, \ldots, \alpha_{J-1}, \sigma^2\}$ while for piecewise linear models (M_{linear} models), $\theta = \{\alpha_0, \beta_0 \ldots, \alpha_{J-1}, \beta_{J-1}, \sigma^2\}$. Let $L_J(\mathbf{s}, \mathbf{x}; \hat{\theta})$ denote the likelihood of the segmentation $\mathbf{s}$ in J segments of the observed series $\mathbf{x}$. The estimation of the $J-1$ change points $\tau_1, \ldots, \tau_{J-1}$, which corresponds to the optimal segmentation $\mathbf{s}^*$ into J segments, is obtained as follows

$$\hat{\tau}_1, \ldots, \hat{\tau}_{J-1} = \arg\max_{\mathbf{s}} \log L_J(\mathbf{s}, \mathbf{x}; \hat{\theta}),$$

with

$$\log L_J(\mathbf{s}, \mathbf{x}; \hat{\theta}) = -\frac{T}{2}\left[\log\left\{\frac{\sum_{j=0}^{J-1}\sum_{t=\tau_j}^{\tau_{j+1}-1}(x_t - \hat{\alpha}_j)^2}{T}\right\} + \log(2\pi) + 1\right] \qquad \text{for } M_{\text{constant}} \text{ model,}$$

$$\log L_J(\mathbf{s}, \mathbf{x}; \hat{\theta}) = -\frac{T}{2}\left[\log\left\{\frac{\sum_{j=0}^{J-1}\sum_{t=\tau_j}^{\tau_{j+1}-1}(x_t - \hat{\alpha}_j - \hat{\beta}_j t)^2}{T}\right\} + \log(2\pi) + 1\right] \qquad \text{for } M_{\text{linear}} \text{ model.}$$

For this optimization task, the additivity in j of the sum of squared deviations, allows us to use a dynamic programming algorithm (Auger and Lawrence, 1989) whose computational complexity is $O(JT^2)$ in time.

Once a multiple change-point model has been estimated for a fixed number of segments J, the question is then to choose this number. In a model selection context, the purpose is to estimate J by maximizing a penalized version of the log-likelihood defined as follows

$$\hat{J} = \arg\max_{J \geq 1} \left\{\log L_J(\mathbf{s}^*, \mathbf{x}; \hat{\theta}) - \text{Penalty}(J)\right\}.$$

The principle of this kind of penalized likelihood criterion consists in making a trade-off between an adequate fitting of the model to the data (expressed by the log-likelihood) and a reasonable number of parameters to be estimated (controlled by the penalty term). The most popular information criteria such as AIC and BIC are not adapted in this particular context since they tend to underpenalize the log-likelihood and thus select a too large number of segments. Zhang and Siegmund (2007) proposed a modified BIC criterion in the case of M_{constant} model. This criterion is given by

$$\text{mBIC}_J = 2\log L_J(\mathbf{s}^*, \mathbf{x}; \hat{\theta}) - 2J\log T - \sum_{j=0}^{J-1} \log(\hat{\tau}_{j+1} - \hat{\tau}_j),$$

where

$$\begin{aligned}\min_{0<\tau_1<\cdots<\tau_{J-1}<T} \sum_{j=0}^{J-1} \log(\hat{\tau}_{j+1} - \hat{\tau}_j) &= \log(T - J + 1) \\ &\approx J\log T - (J-1)\log T \quad \text{if } J \ll T, \\ \max_{0<\tau_1<\cdots<\tau_{J-1}<T} \sum_{j=0}^{J-1} \log(\hat{\tau}_{j+1} - \hat{\tau}_j) &= J\log\frac{T}{J} \\ &= J\log T - J\log J.\end{aligned}$$

Hence each change point contributes between 1 and 2 dimensions to the penalty term (instead of systematically 1 dimension for each within-segment or global variance parameter) and this penalty term is maximized when the change points are evenly spaced. For M_{linear} model, mBIC is given by

$$\text{mBIC}_J = 2\log L_J(\mathbf{s}^*, \mathbf{x}; \hat{\theta}) - 3J\log T - \sum_{j=0}^{J-1} \log(\hat{\tau}_{j+1} - \hat{\tau}_j),$$

The posterior probability of the *J*-segment model M_J, given by

$$P(M_J \mid \mathbf{x}) = \frac{\exp\left(\frac{1}{2}\text{mBIC}_J\right)}{\sum_{k=1}^{J_{\max}} \exp\left(\frac{1}{2}\text{mBIC}_K\right)},$$

can be used to assess the relative merits of the models considered.

References

Auger, I. E., and Lawrence, C. E. (1989). Algorithms for the optimal identification of segment neighborhoods. *Bull. Math. Biol*. 51, 39-54.

Zhang, N. R., and Siegmund, D. O. (2007). A modified Bayes information criterion with applications to the analysis of comparative genomic hybridization data. *Biometrics* 63, 22-32.

Table S1. Standard deviations estimated in 2-segment piecewise constant models for sufficiently long (initial date ≤ 1975) flowering date series (BBCH 61 stage for all locations except Conthey – BBCH 65 stage)

	Cultivar	Segment 1	Standard deviation 1	Segment 2	Standard deviation 2
Angers	Golden D.	1963–1988	6.98	1989–2013	6.79
Nîmes	Golden D.	1974–1988	6.43	1989–2013	7.38
Forli	Golden D.	1970–1987	7.09	1988–2013	6.71
Bonn	Golden D.	1958–1988	8.8	1989–2013	6.75
Conthey (65)	Golden D.	1970–1987	6.4	1988–2013	4.7
Conthey (65)	Gala	1975–1987	5.46	1988–2013	5.15

Table S2. Standard deviations estimated in 2-segment piecewise constant models for sufficiently long series of mean temperatures during the HA period (initial date ≤ 1970)

	Segment 1	Standard deviation 1	Segment 2	Standard deviation 2
Angers	1963–1986	0.93	1987–2013	0.95
Nîmes	1966–1987	0.94	1988–2013	0.88
Forli	1970–1987	0.88	1988–2013	0.95
Gembloux	1964–1988	1.12	1989–2013	1.19
Bonn	1959–1988	1.34	1989–2013	1.16
Conthey	1970–1987	1.18	1988–2013	0.96

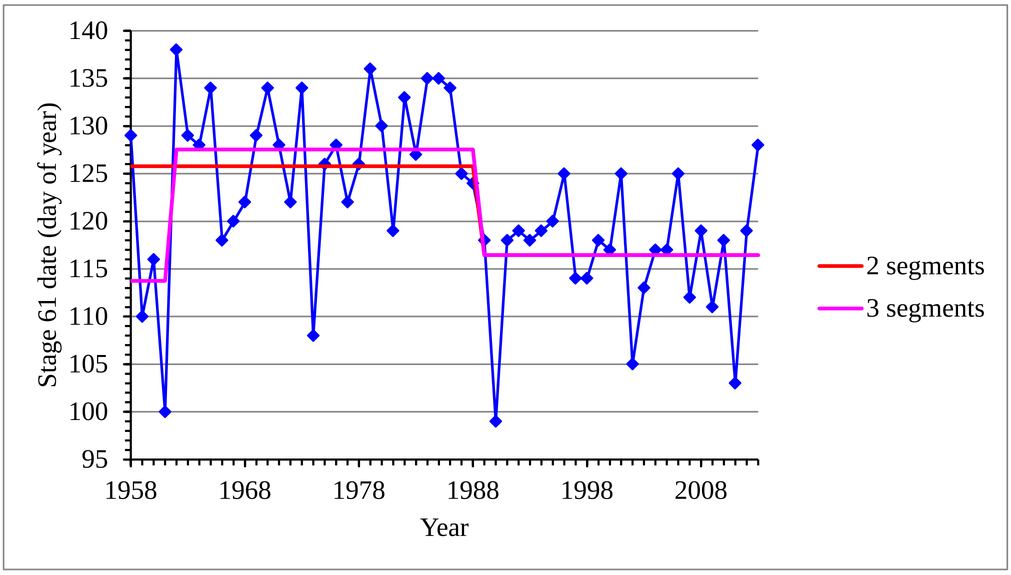

Figure S1. Segmentation of the Bonn BBCH 61 stage date series using 2-and 3-segment piecewise constant models
